# Regulation of Transcription Factor Yin Yang 1 by SET7/9-mediated Lysine Methylation

**DOI:** 10.1038/srep21718

**Published:** 2016-02-23

**Authors:** Wen-juan Zhang, Xiao-nan Wu, Tao-tao Shi, Huan-teng Xu, Jia Yi, Hai-feng Shen, Ming-feng Huang, Xing-yi Shu, Fei-fei Wang, Bing-ling Peng, Rong-quan Xiao, Wei-wei Gao, Jian-cheng Ding, Wen Liu

**Affiliations:** 1 School of Pharmaceutical Sciences, Xiamen University, Xiang’an South Road, Xiamen, Fujian 361102, China; 2 College of Chemistry and Chemical Engineering, Xiamen University, No. 422 Siming South Road, Xiamen, Fujian 361105, China

## Abstract

Yin Yang 1 (YY1) is a multifunctional transcription factor shown to be critical in a variety of biological processes. Although it is regulated by multiple types of post-translational modifications (PTMs), whether YY1 is methylated, which enzyme methylates YY1, and hence the functional significance of YY1 methylation remains completely unknown. Here we reported the first methyltransferase, SET7/9 (KMT7), capable of methylating YY1 at two highly conserved lysine (K) residues, K173 and K411, located in two distinct domains, one in the central glycine-rich region and the other in the very carboxyl-terminus. Functional studies revealed that SET7/9-mediated YY1 methylation regulated YY1 DNA-binding activity both *in vitro* and at specific genomic loci in cultured cells. Consistently, SET7/9-mediated YY1 methylation was shown to involve in YY1-regulated gene transcription and cell proliferation. Our findings revealed a novel regulatory strategy, methylation by lysine methyltransferase, imposed on YY1 protein, and linked YY1 methylation with its biological functions.

YY1 is a ubiquitous and multifunctional zinc-finger transcription factor that is involved in a variety of biological processes, including development, cell proliferation and differentiation, DNA repair, and apoptosis, among others^1^,^2^,^3^,^4^,^5^,^6^,^7^,^8^,^9^. YY1 is essential for the development of mouse embryo, with ablation of *yy1* in mice resulting in embryonic lethality. Specifically, *yy1* mutants undergo implantation and induce uterine decidualization but rapidly degenerate around the time of implantation, and *yy1* heterozygote embryos display severe developmental abnormalities^10^. Interestingly, mouse embryonic fibroblast (MEF) cells from mice carrying *yy1* alleles expressing various amounts of YY1 display a dosage-dependent requirement of YY1 for cell proliferation^11^. Accordingly, inhibition of YY1 in cultured cells leads to cytokinesis defects and cell cycle arrest^11^. YY1 was also shown to function in homologous recombination-based DNA repair (HRR), presumably through its interaction with INO80 chromatin-remodeling complex^12^. The role of YY1 in apoptosis was first suggested based on the observation that YY1 negatively regulates Hdm2-mediated p53 degradation^13^. Moreover, YY1 itself is cleaved by caspases both *in vitro* and *in vivo* in response to apoptotic stimuli. The cleaved YY1 product, but not wild-type protein can modify the apoptotic response to anti-Fas, suggesting that cleaved YY1 plays a positive feedback role during later stages of apoptosis^14^. Ample studies indicate expression of YY1 is deregulated in different cancers, including prostate cancer, breast cancer, ovarian cancer, brain cancer, osteosarcoma, colon cancer, cervical cancer, large B-cell and follicular lymphoma, acute myeloid leukemia, and hepatoblastoma^1^,^2^,^4^,^5^.

YY1 exerts its biological functions primarily as a sequence-specific DNA binding transcription factor that can activate or repress gene expression. The structural and functional domains of YY1 protein have been well characterized^15^,^16^,^17^. It contains a transactivation domain at its amino-terminus, a repression domain at its central portion, and a DNA binding domain constituted of four zinc fingers of the C2H2 type at its carboxyl-terminus. All four fingers have been shown to be required for proper binding to DNA and involved in transcriptional regulation.

Numerous mechanisms have been shown to regulate the function of YY1, such as its associated co-factors, subcellular localization, post-translational modifications including poly(ADP-ribosyl)ation, ubiquitination, acetylation, O-linked glycosylation, S-nitrosation, sumoylation and phosphorylation. YY1 has been shown to be poly(ADP-ribosyl)ated under genotoxic stress, which negatively regulates its affinity with its DNA binding sites^18^. In 1998, Walowitz *et al.* demonstrated that YY1 is a substrate for ubiquitination^19^. However the exact lysine residues modified by ubiquitination were not determined. Recently, several global proteomic studies have revealed multiple ubiquitination sites including lysine 258^20^, 174, 203, 204, 339 and 369 (Cell Signaling Technology), with the enzymes responsible for and the function of these modifications remaining to be explored. More recently, Smurf2 was shown to act as an E3 ubiquitin ligase mediating YY1 ubiquitination and degradation, which suppresses B-cell proliferation and lymphomagenesis^21^,^22^. Two histone acetyltransferases (HATs), p300 and PCAF (p300-CBP associated factor), have been shown to acetylate YY1 at its central region, which is required for its fully transcriptional repressor activity. PCAF also acetylates YY1 at its C-terminal DNA-binding domain, which might decrease its DNA binding activity^23^. In response to glucose stimulation, YY1 is O-GlcNAcylated and glycosylated YY1 is released from the Rb protein and free to bind DNA^24^. Nitric oxide (NO)-induced YY1 S-nitrosylation inhibits its DNA-binding activity, with a functional implication in tumor cell sensitization to Fas-induced apoptosis^25^. PIASy, a SUMO E3 ligase, has been shown to sumoylate YY1, which increases its stability and represses its transcriptional activity^26^. Recently, it was shown that the phosphorylation level of YY1 increased dramatically in mitotic cells, which correlates the loss of YY1 DNA-binding activity in mitosis. Furthermore, three phosphorylation sites, serine 247 (S247), threonine 348 (T348) and 378 (T378), were identified, with T348 and T378 phosphorylation proving to be essential for DNA-binding activity of YY1 *in vitro*^27^. The same group then identified the first three kinases capable of phosphorylating YY1. Specifically, Polo-like kinase 1 (Plk1) was shown to phosphorylate YY1 at threonine 39 (T39) both *in vitro* and *in vivo*. T39 phosphorylation is regulated during cell cycle and peaks at G2/M^28^. Casein kinase 2α (CK2α) was the second kinase shown to phosphorylate YY1 at serine 118 (S118) in the transactivation domain of YY1. Functional study revealed that blocking S118 phosphorylation increases the cleavage of YY1 during apoptosis^29^. More recently, it was reported that Aurora B kinase phosphorylates YY1 in the central glycine/alanine (G/A)-rich region at serine 184 (S184) both *in vitro* and *in vivo*, which peaks at G2/M and is rapidly dephosphorylated as the cells enters G1. Importantly, phosphorylation of YY1 at S184 is important for its DNA binding activity^30^. Despite it has been shown to associate with multiple histone methyltransferases, such as protein arginine N-methyltransferase 1 (PRMT1)^31^, enhancer of zest homologue 1 (EZH1)^32^ and enhancer of zest homologue 2 (EZH2)^33^, methylation of YY1, the enzymes responsible for such methylation, and hence its function have not been reported so far.

SET7/9 was initially identified as a histone lysine methyltransferase which modifies histone H3 lysine 4^34^,^35^. However, it exhibited very weak activity when nucleosomes were used as substrates, implying that non-histone proteins might be the cogent targets through which SET7/9 exerts its function^34^. Indeed, since the discovery of p53 methylation by SET7/9, an array of non-histone proteins were shown to be targeted by SET7/9, including nuclear receptors, chromatin-associated transcriptional factors and co-factors, and chromatin-modifying enzymes, among others. Accordingly, SET7/9 has been implicated in a wide range of cellular functions^36^,^37^. Based on crystal structure, it has been suggested that SET7/9 mainly recognizes the sequence motif [K/R] [S/T/A] K (in which the methylation site is underlined) in its targets^38^. More recently, peptide array was applied to determine an optimized target sequence for SET7/9, which revealed multiple additional non-histone substrates^39^.

Here we presented evidences that SET7/9 methylated YY1 both *in vitro* and *in vivo*, and SET7/9-mediated YY1 methylation regulated its DNA-binding activity. Gro-seq (global run-on coupled to high throughput sequencing) analysis revealed that a substantial list of genes was regulated by YY1, many of which were implicated in regulation of cell proliferation. Consistently, SET7/9-mediated YY1 methylation was shown to be important for YY1-regulated cell proliferation.

## Results

### YY1 is methylated by SET7/9 *in vitro*

**Figure 1:**
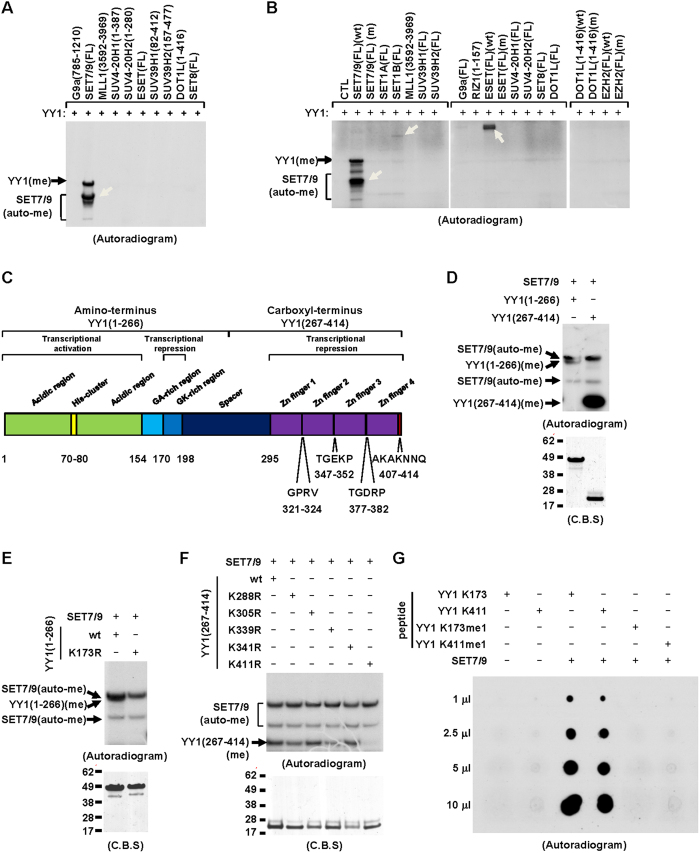
YY1 is methylated by SET7/9 *in vitro*. (**A,B**) *In vitro* methylation assay was performed by mixing purified bacterially-expressed His-tagged YY1 protein with various protein lysine methyltransferases (KMTs), either full length (FL) or truncations with enzymatic domain, from bacterial cells (**A**) or HEK293T cells with over-expression (**B**) as indicated, followed by autoradiogram. Wild-type: wt; Enzymatically dead mutant: m. White arrows indicate automethylation (auto-me) of KMTs; Black arrows indicate methylation of YY1 (YY1(me)). (**C**) Schematic representation of the domain architecture of YY1 protein. Amino acid information for the three linker regions, aa321–324, 347–352 and 377–382, between Zn fingers as well as the very carboxyl-terminus was depicted. Acidic Region (light green); His-cluster (yellow); GA-rich region (light blue); GK-rich region (blue); Spacer (dark blue); Zn finger (purple); Linker region (black); The very carboxyl-terminus (red). (**D**) *In vitro* methylation assay was performed by mixing purified bacterially-expressed His-tagged SET7/9 protein with YY1 amino-terminus (1-266) or carboxyl-terminus (267-414), followed by autoradiogram (top panel). The expression of YY1(1-266) and YY1(267-414) was examined by coomassie blue staining (C.B.S) (bottom panel). (**E**) *In vitro* methylation assay was performed by mixing purified bacterially-expressed His-tagged SET7/9 with YY1(1-266) wild type (wt) or its mutant form with substitution of lysine 173 to arginine (K173R), followed by autoradiogram (top panel). The expression of YY1(1-266)(wt) and K173R was examined by C.B.S (bottom panel). (**F**) *In vitro* methylation assay was performed by mixing purified bacterially-expressed His-tagged SET7/9 with YY1(267-414) wild type (wt) or its mutant form with substitution of lysine 288, 305, 339, 341 or 411 to arginine (K288R, K305R, K339R, K341R or K411R), followed by autoradiogram (top panel). The expression of YY1(267-414)(wt), K288R, K305R, K339R, K341R and K411R was examined by C.B.S (bottom panel). (**G**) *In vitro* methylation assay was performed by mixing short peptides containing unmodified (K173 or K411) or mono-methylated K173 or K411 (K173me1 or K411me1) with or without purified bacterially-expressed SET7/9 proteins. Increased amount of each reaction was taken for dot blot as indicated, followed by autoradiogram.

YY1 was shown to interact with multiple histone methyltransferases, which raised the possibility that YY1 might be a direct target for methylation. We tested this possibility by performing *in vitro* methylation assay mixing purified bacterially-expressed YY1 with several histone lysine methyltransferases known to target to histone H3 or H4. It was found that YY1 was robustly methylated by SET7/9 (Fig. 1A). Meanwhile, auto-methylation of SET7/9 was also observed (Fig. 1A). Of note, many of the enzymes tested displayed no activity when core histones were serving as substrates under current conditions (Supplementary Fig. 1A). The expression of all enzymes tested was shown by coomassie blue staining (C.B.S) (Supplementary Fig. 1B). To further test YY1 methylation by histone lysine methyltransferase, we included several additional enzymes, and purified all these enzymes from over-expressed HEK293T cells. Once again, it was found that wild-type (wt) SET7/9, but not its enzymatically dead mutant (m) or other methyltransferases tested, methylated YY1 (Fig. 1B). Interestingly, besides SET7/9, several other methyltransferases, including SET1B, G9a and ESET, also exhibited auto-methylation activity when purified from over-expressed HEK293T cells (Fig. 1B and Supplementary Fig. 1C). Of note, enzymes except EZH2 were shown to be active when core histones were serving as substrates under current conditions (Supplementary Fig. 1D). The expression of all enzymes tested was shown by immunoblotting using antibodies as indicated (Supplementary Fig. 1E). We focused on studying YY1 methylation by SET7/9 in the current study, and meanwhile do not rule out the possibility that other enzymes might also be able to methylate YY1 under different experimental conditions.

Next, we sought to identify the lysine residues in YY1 targeted by SET7/9. Firstly, *in vitro* methylation assay was performed by mixing purified bacterially-expressed SET7/9 and YY1 truncations encompassing amino-terminal (aa1–266) or carboxyl-terminal (aa267–414) of YY1, finding that both truncations were methylated by SET7/9 (Fig. 1C,D). Previous structural study and peptide array revealed a consensus peptide sequence targeted by SET7/9: [K > R] [S > KYARTPN] K (in which the methylation site is underlined)^38^,^39^. Examination of YY1 sequence revealed that there are six putative lysine methylation sites, either consensus or degenerative, located in YY1, with one (lysine 173) at the amino- and five (lysine 288, 305, 339, 341 and 411) at the carboxyl-terminal of YY1, respectively (Supplementary Fig. 1F). Replacing lysine 173 with arginine (K173R) abolished SET7/9-mediated methylation of YY1 at its amino-terminus (Fig. 1E). Similarly, mutation of lysine 411 (K411R) completely blocked YY1 methylation mediated by SET7/9 at its carboxyl-terminus, whereas the other mutations, including K288R, K305R, K339R and K341R, displayed no dramatic effects (Fig. 1F). The methylation of lysine 173 and 411 by SET7/9 was further confirmed by using short peptides from YY1 containing these lysines (Fig. 1G). Moreover, SET7/9 was found to mono-methylate K173 and K411 as short peptides containing mono-methylated K173 (K173me1) or K411 (K411me1) no longer served as substrates for SET7/9 (Fig. 1G). Importantly, both lysine 173 and 411 were found to be highly conserved during evolution, suggesting methylation on these residues might be functionally important (Supplementary Fig. 1G and 1H).

### YY1 is methylated by SET7/9 in cultured cells

**Figure 2:**
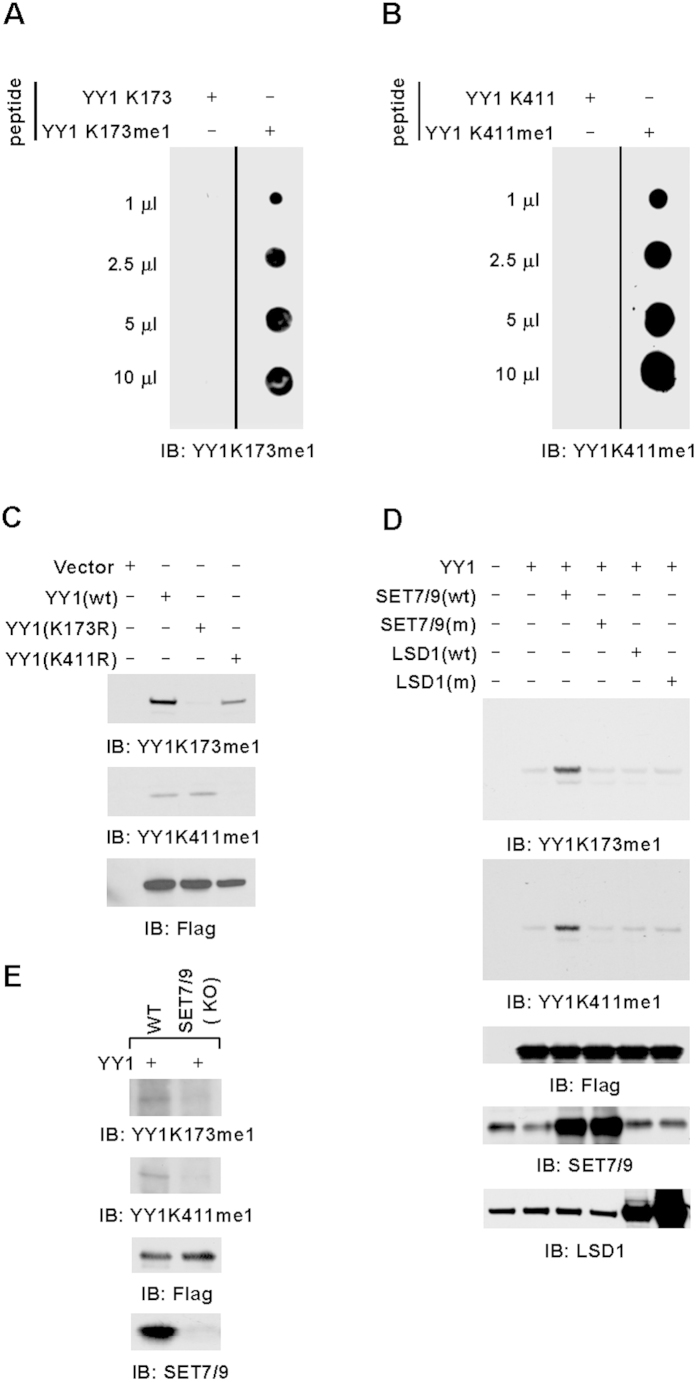
YY1 is methylated by SET7/9 in cultured cells. (**A,B**) Increased amount of short peptides containing unmodified (K173 or K411) or mono-methylated K173 or K411 (K173me1 or K411me1) were prepared for dot blot assay, followed by immunoblotting (IB) using anti-YY1K173me1 (**A**) or anti-YY1K411me1 (**B**) antibody as indicated. (**C**) HEK293T cells transfected with control vector or vectors expressing Flag-tagged wild type (wt) or mutant YY1 (K173R or K411R) were subjected to IB with anti-YY1K173me1, anti-YY1K411me1 or anti-Flag antibody as indicated. (**D**) HeLa cells transfected with control vector or vector expressing Flag-tagged YY1 in the presence or absence of SET7/9, LSD1 wild type (wt) or enzymatically dead mutant (m) were subjected to IB with anti-YY1K173me1, anti-YY1K411me1, anti-Flag, anti-SET7/9 and anti-LSD1 antibody as indicated. (**E**) Wild type or SET7/9 knock-out (KO) HeLa cells were transfected with Flag-tagged YY1 followed by immunoblotting (IB) with antibodies as indicated.

To examine whether SET7/9 methylates YY1 in cultured cells and the functional importance of this methylation, antibodies specifically targeting mono-methylated lysine 173 (anti-K173me1) or 411 (anti-K411me1) were generated. The specificity of these antibodies was tested in multiple ways. Firstly, short peptides containing unmodified or mono-methylated K173 were subjected to dot blotting with anti-K173me1 antibody. It was found that anti-K173me1 antibody only recognized peptide containing mono-methylated K173, but not unmodified K173 (Fig. 2A). Similarly, anti-K411me1 was shown to recognize mono-methylated K411 only (Fig. 2B). Secondly, total lysates from HEK293T cells transfected with a control vector or vectors expressing Flag-tagged wild type YY1(wt) or YY1 mutants with substitution of lysine 173 or 411 to arginine (K173R or K411R) were subjected to immunoblotting (IB) with anti-K173me1 or anti-K411me1 antibody. It was found that, when cells were transfected with YY1(wt), both anti-K173me1 and anti-K411me1 antibodies detected a specific band, which presumably corresponded to YY1 protein (Fig. 2C). As expected, mutation of K173 or K411 completely abolished the chemiluminescent signals detected by anti-K173me1 or anti-K411me1 antibody, respectively, further confirming the specificity of these two antibodies (Fig. 2C). The expression of YY1(wt), YY1(K173R) and YY1(K411R) was examined using anti-Flag antibody (Fig. 2C). Of note, both anti-K173me1 and anti-K411me1 antibodies failed to detect endogenous YY1 proteins under current conditions.

Taking advantage of the anti-K173me1 and anti-K411me1 antibodies generated, we then tested whether SET7/9 methylates YY1 in cultured cells by co-transfecting HeLa cells with YY1 and wild type SET7/9 (wt) or its enzymatically dead mutant (m) followed by IB with anti-K173me1 or anti-K411me1 antibody. As expected, wild type SET7/9, but not its enzymatically dead mutant, resulted in a significant increase of both K173 and K411 methylation levels (Fig. 2D). To our surprise, knock-down of SET7/9 did not influence either K173 or K411 methylation levels globally, which could be due to that other lysine methyltransferases yet to be discovered compensate the loss of SET7/9 (Supplementary Fig. 2A). It is also well-known that, for some of the epigenetic enzymes, such as CARM1, residual proteins after siRNA-mediated knock-down will still be fully functional in terms of its enzymatic activity^40^. Therefore, CRISPR/Cas9-mediated gene editing technology was applied to generate SET7/9 knock-out HeLa cell lines (Supplementary Fig. 2B). To our surprise, knocking out of SET7/9 led to a nearly complete loss of K173 and K411 methylation (Fig. 2E). We concluded that SET7/9 was mainly, if not solely, responsible for K173 and K411 methylation in cultured cells. As LSD1, the first discovered lysine demethylase, has opposing activity to SET7/9 on histone as well as several non-histone proteins^41^,^42^,^43^, HeLa cells were transfected with YY1 and wild type LSD1 (wt) or its enzymatically dead mutant (m) to see whether LSD1 targets SET7/9-mediated YY1 methylation at K173 and/or K411. Surprisingly, co-transfection of LSD1 displayed no significant effects on methylation on either lysine residue (Fig. 2D). Similarly, knocking down of LSD1 in HeLa cells had no significant impact on SET7/9-mediated YY1 methylation (Supplementary Fig. 2A).

### SET7/9-mediated YY1 methylation regulates YY1 DNA-binding activity

**Figure 3:**
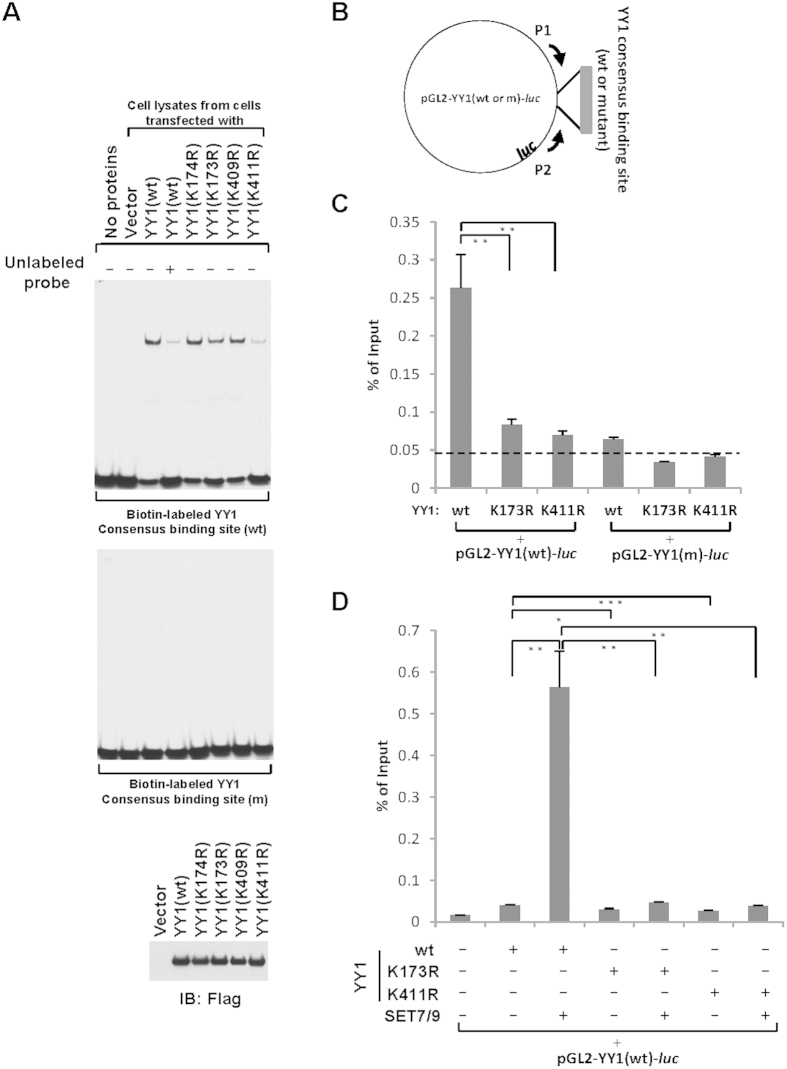
SET7/9-mediated YY1 methylation regulates YY1 DNA-binding activity. (**A**) DNA EMSA assay was performed by incubating biotinylated oligonucleotide containing YY1 consensus binding site (wt) (top panel) or its mutant form (m) (middle panel) with or without whole cell lysates prepared from HEK293T cells transfected with control vector or vectors expressing Flag-tagged YY1(wt), YY1(K173R), YY1(K174R), YY1(K409R) or YY1(K411R). Unlabeled oligonucleotide was included as indicated to demonstrate the specificity of YY1 binding with its consensus binding site. The binding affinity (gel intensity) was quantified by using Image J, with the ratio of lane 3:4:5:6:7:8 being 1:0.15:0.94:0.62:0.85:0.2. The expression of YY1(wt), YY1(K173R), YY1(K174R), YY1(K409R) and YY1(K411R) was examined through IB with anti-Flag antibody. (**B**) YY1 consensus binding site or its mutant form was cloned into pGL2-luciferase vector (pGL2-YY1(wt)-*luc* or pGL2-YY1(m)-*luc*). YY1 binding to the consensus binding site or its mutant form can be examined through ChIP using a primer set specifically targeting to upstream (P1) or downstream (P2) of multiple cloning site in pGL2 vector. (**C**) HeLa cells were transfected with pGL2-YY1(wt)-*luc* or pGL2-YY1(m)-*luc* vector in the presence or absence of vectors expressing Flag-tagged YY1(wt), YY1(K173R) or YY1(K411R), followed by ChIP with anti-Flag antibody and then q-PCR with the primer set (P1 + P2) as described in (**B**). ChIP signals were presented as percentage of inputs (±s.e.m., **P < 0.01). Experiments were repeated three times and representative data was shown. (**D**) HeLa cells were transfected with pGL2-YY1(wt)-*luc* vector together with or without Flag-tagged YY1(wt), YY1(K173R) or YY1(K411R) in the presence or absence of SET7/9, followed by ChIP with anti-Flag antibody and q-PCR with the primer set (P1 + P2) as described in (B). ChIP signals were presented as percentage of inputs (±s.e.m., *P < 0.05, **P < 0.01, ***P < 0.001). Experiments were repeated three times and representative data was shown.

YY1 is a ubiquitously expressed zinc finger DNA binding protein, which binds to a consensus DNA element containing a conserved 5′-CAT-3′ core flanked by variable regions (5′-(C/g/a)(Gtt)(Ctt/a)CATN(T/a)(T/g/c)-3′)^44^,^45^. To examine whether SET7/9-mediated YY1 K173 and K411 methylation regulates its DNA-binding activity, electrophoretic mobility shift assay (EMSA) was performed by mixing a biotinylated oligonucleotide containing YY1 consensus binding site with whole cell extracts collected from HEK293T cells transfected with a control vector or vectors expressing wild type YY1(wt) or YY1 mutants with substitution of lysine 173, 174, 409 or 411 to arginine (K173R, K174R, K409R or K411R). It was found that incubation with wild type YY1, but not the control vector, led to a specific shift of the biotinylated oligonucleotide containing YY1 consensus binding site, which was inhibited by adding unlabeled oligonucleotide (Fig. 3A, upper panel, lane 1 to 4). Moreover, an oligonucleotide with a mutated YY1 binding site completely abolished such shift, further supporting the specificity of the observed YY1 binding with its consensus site by EMSA (Fig. 3A, middle panel). Surprisingly, YY1 mutants, K173R and K411R, displayed a much weaker binding (less shift) compared to wild type YY1 (Fig. 3A, upper panel, compared lane 6 and 8 to 3). Importantly, two lysine residues K174 and K409, adjacent to K173 and K411, respectively, were substituted to arginine (K174R and K409R), which had no significant impact on YY1 binding affinity with its consensus binding site, suggesting the decreased DNA-binding affinity of YY1(K173R) and YY1(K411R) was most likely due to a post-translational modification on these sites, rather than an overall structural change on YY1 (Fig. 3A, upper panel, compared lane 5 and 7 to 3). Wild type and all mutant YY1 were expressed equally well as assessed by IB (Fig. 3A, bottom panel).

To further demonstrate YY1 binding with DNA is regulated by SET7/9-mediated methylation, firstly, HeLa cells were transfected with luciferase reporter vectors containing YY1 consensus binding site (pGL2-YY1(wt)-*luc*) or its mutant form (pGL2-YY1(m)-*luc*), and vectors expressing Flag-tagged YY1(wt), YY1(K173R) or YY1(K411R), followed by chromatin immunoprecipitation (ChIP) assay with anti-Flag antibody (Fig. 3B,C). As expected, YY1 displayed much stronger binding on its consensus binding site compared to the mutant form examined through q-PCR using a primer set (P1 + P2) specifically targeting to the luciferase vector (Fig. 3B,C, compared bar 1 to 4). More importantly, K173R and K411R significantly impaired YY1 DNA-binding activity compared to YY1(wt) (Fig. 3C, compared bar 2 and 3 to 1). Secondly, HeLa cells were transfected with pGL2-YY1(wt)-*luc* vector together with or without Flag-tagged YY1(wt), YY1(K173R) or YY1(K411R) in the presence or absence of SET7/9, followed by ChIP with anti-Flag antibody (Fig. 3D). Consistent with the observation that SET7/9 induced YY1 methylation, co-transfection of SET7/9 led to a significant increase of Flag-tagged YY1 binding with YY1 consensus site (compare bar 2 to 3), which was attenuated when K173 or K411 was mutated (compare bar 3 to 5 and 7), suggesting SET7/9-induced YY1 binding was dependent on K173 and K411 (Fig. 3D).

### SET7/9-mediated YY1 methylation regulates YY1 genomic association

**Figure 4:**
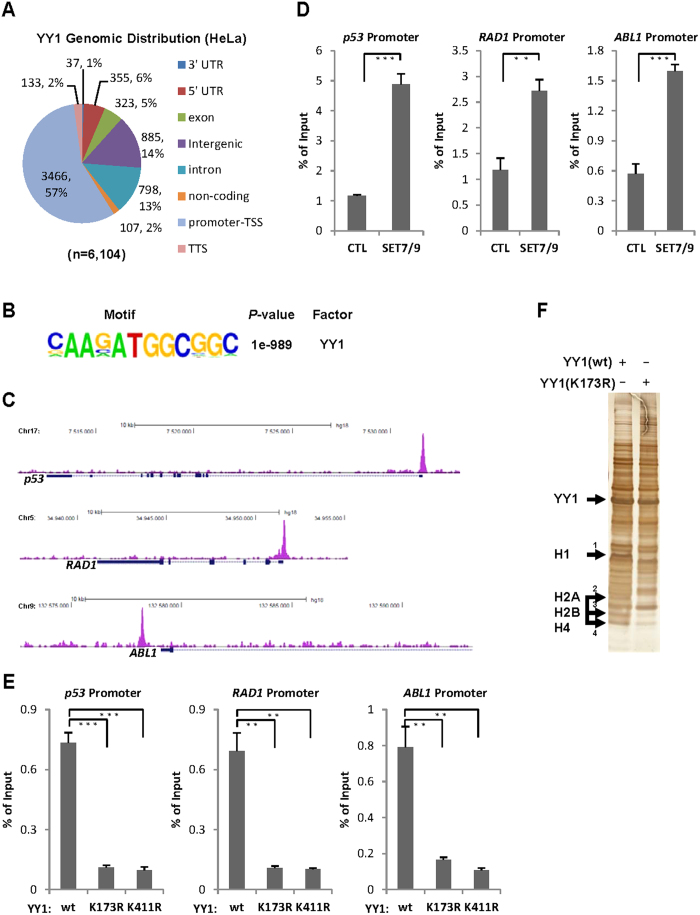
SET7/9-mediated YY1 methylation regulates YY1 genomic association. (**A,B**) Genomic distribution of YY1 binding sites. ChIP-seq was performed in HeLa cells using anti-YY1 antibody, followed by peak finding (**A**) and motif analysis (**B**) using HOMER. (**C**) YY1 binding detected by ChIP-seq was shown for *p53*, *RAD1* and *ABL1* genes, as indicated. (**D**) HeLa cells were transfected with control vector or vector expressing SET7/9, followed by ChIP with anti-YY1 antibody and q-PCR with primers specifically targeting promoter regions of selected genes as indicated. ChIP signals were presented as percentage of inputs (±s.e.m., **P < 0.01, ***P < 0.001). Experiments were repeated three times and representative data was shown. (**E**) HeLa cells were transfected with vectors expressing Flag-tagged YY1(wt), YY1(K173R) or YY1(K411R), followed by ChIP with anti-Flag antibody and q-PCR with primers specifically targeting promoter regions of selected genes as indicated. ChIP signals were presented as percentage of inputs (±s.e.m., **P < 0.01, ***P < 0.001). Experiments were repeated three times and representative data was shown. (**F**) HeLa cells stably expressing Flag-tagged YY1(wt) or YY1(K173R) were subjected to affinity purification with Flag M2 agarose. The resultant proteins were separated by SDS-PAGE gel, followed by silver staining. Four bands (1–4) specifically present in YY1(wt) sample were cut and subjected to mass spectrometry (MS) analysis.

To test whether SET7/9-mediated YY1 methylation regulates YY1 binding on its associated genomic regions, YY1 ChIP-seq (chromatin immunoprecipitation coupled with high throughput sequencing) in HeLa cells was performed using a specific antibody against YY1. It was found that around 57% of YY1 binding sites located on gene promoter regions (transcription start site, TSS), whereas the rest 43% on non-promoter regions, including 3′ UTR (untranslated region), 5′ UTR, exon, intron, TTS (transcription termination site), intergenic and non-coding RNA regions (Fig. 4A). Motif analysis revealed that the most enriched DNA motif in all YY1 binding sites identified indeed fully matched to the reported YY1 consensus binding motif, suggesting the YY1 ChIP-seq experiment was valid (Fig. 4B). YY1 binding detected by ChIP-seq was shown on some selected promoters, such as *p53*, *RAD1* and *ABL1* (Fig. 4C). To test whether SET7/9 regulates YY1 binding on specific gene loci, HeLa cells were transfected with control or SET7/9 expression vector, followed by ChIP with anti-YY1 antibody. It was found that YY1 binding increased significantly on the selected gene promoter regions, including *p53*, *RAD1*, *ABL1*, *CCND1, CCNT2* and *CCNA2* upon SET7/9 transfection (Fig. 4D and Supplementary Fig. 3A). Despite lacking of global effects on YY1 K173 or K411 methylation levels, knock-down of SET7/9 led to a specific and significant decrease of YY1 binding on the promoter regions of those genes tested above (Supplementary Fig. 3B). Of note, SET7/9 effects on YY1 binding seemed to be loci specific as it did not influence YY1 binding on some other genomic loci tested (Supplementary Fig. 3C). Unaltered YY1 binding upon SET7/9 knock-down on some genes’ promoter regions might be due to lack of co-binding of SET7/9, reflecting regulation of YY1 binding by its associated factors in a gene specific manner.

To test whether K173 and K411 regulates YY1 binding, HeLa cells were transfected with vectors expressing Flag-tagged YY1(wt), YY1(K173R) or YY1(K411R) followed by ChIP assay with anti-Flag antibody. It was found that binding of YY1(K173R) and YY1(K411R) was significant lower compared to YY1(wt) (Fig. 4E and Supplementary Fig. 3D). To further test the requirement of K173 and K411 in YY1 binding with chromatin, HeLa cells stably expressing Flag-tagged YY1(wt) or YY1(K173R) were subjected to affinity purification with anti-Flag antibody, and the interacting proteins were resolved by SDS-PAGE gel and followed by silver staining. To our surprise, several distinct bands were specifically present in the YY1(wt) interactome, which were then identified to be histone H1 (Band 1), H2A, H2B and H4 (Band 2, 3 and 4) by mass spectrometry (MS) analysis (Fig. 4F and Supplementary Table 1). YY1(K411R) interactome was also revealed in a similar way, and then compared to that of YY1(wt). It was found that, again, histone H1, H2A, H2B and H4 were specifically present in YY1(wt) but not YY1(K411R) interactome (Supplementary Fig. 3E). It should be noted that histone H3 was not detectable in our MS analysis.

### SET7/9-mediated YY1 methylation is involved in YY1-regulated gene transcriptional program

**Figure 5:**
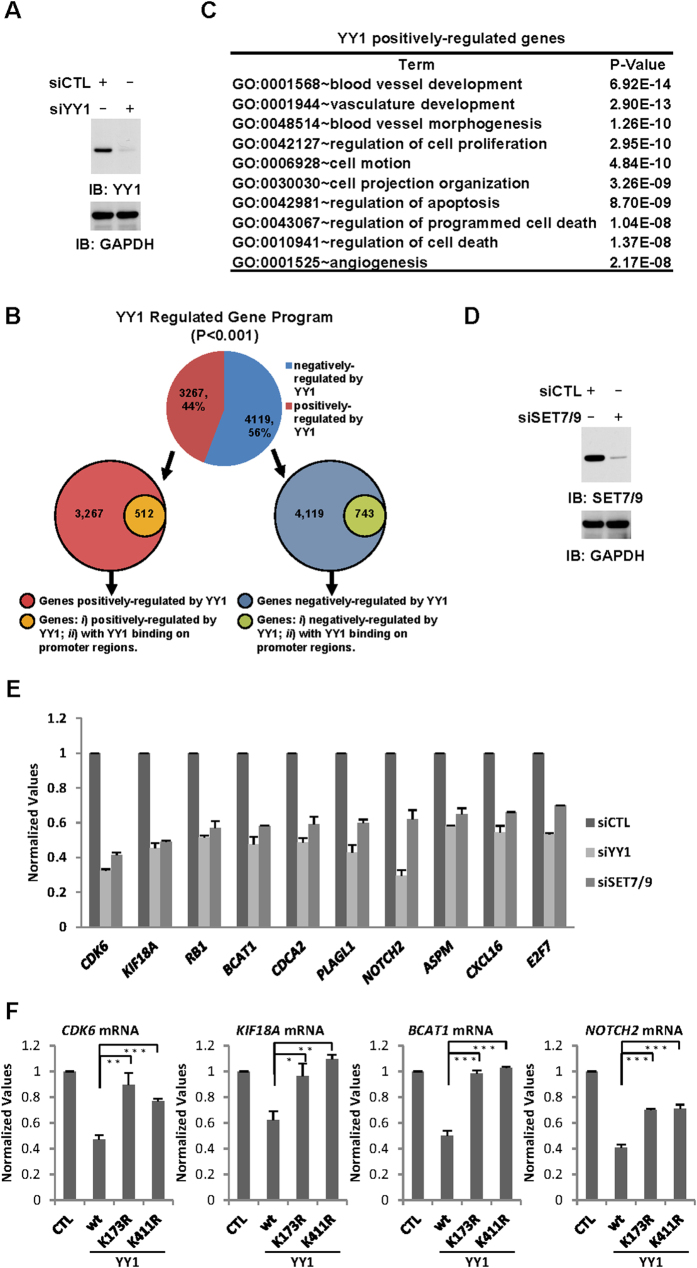
SET7/9-mediated YY1 methylation is involved in YY1-regulated gene transcription. (**A**) HeLa cells were transfected with control siRNA or siRNA specifically targeting *YY1*, followed by immunoblotting using antibodies as indicated to examine the knock-down efficiency of siYY1. (**B**) Gro-seq experiments were performed with nuclei collected from cells described in (A). Genes regulated by YY1 were displayed using pie chart (top, P < 0.001). Among all genes regulated by YY1, those with or without YY1 binding on promoter regions were displayed using Venn diagram (bottom two). (**C**) Gene ontology analysis was performed for genes positively-regulated by YY1 as shown in (B) using DAVID. Top ten enriched gene ontology (GO) terms were shown. (**D**) Knock-down efficiency of siSET7/9 was examined through immunoblotting using antibodies as indicated. (**E**) HeLa cells were transfected with control siRNA or siRNA specifically targeting *YY1* or SET7/9, followed by RT-qPCR analysis to examine mRNA levels of selected genes as indicated. Data shown was the relative fold change compared to control samples after normalization to actin. Experiments were repeated three times and representative data was shown. (**F**) HeLa cells were transfected with control vector or vectors expressing YY1(wt), YY1(K173R) or YY1(K411R), followed by RT-qPCR analysis to examine mRNA levels of selected genes as indicated. Data shown was the relative fold change compared to control samples after normalization to actin (±s.e.m., *P < 0.05, **P < 0.01, ***P < 0.001). Experiments were repeated four times and representative data was shown.

YY1 is a multifunctional zinc-finger transcription factor, which can activate or repress a variety of genes required for development, cell proliferation and differentiation, DNA repair, and apoptosis, among others. It exerts its function primarily through association with chromatin. The observed effects of SET7/9 and YY1 K173/411 on its binding with chromatin prompted us to examine whether SET7/9-mediated YY1 methylation is involved in YY1-regulated gene transcriptional program. Firstly, HeLa cells were transfected with control siRNA or siRNA specifically targeting *YY1* followed by Gro-seq (global run-on coupled with high throughput sequencing) analysis, which detects transcripts generated in nuclear run-on reactions by RNA Pol II that are engaged in and competent for transcription^46^. It was found that 4,119 and 3,267 genes were negatively- and positively-regulated by YY1 (P < 0.001), respectively, confirming it can function as both a repressor and activator in gene transcriptional regulation (Fig. 5A,B). Surprisingly, despite the fact that YY1 largely localized on promoter regions, majority of the YY1-regulated genes exhibited no promoter binding of YY1, suggesting a distal regulatory mechanism underlying its regulated gene transcriptional program, which might be established on distal enhancers or promoters with YY1 binding (Fig. 5B). Consistent with previously reported function of YY1, gene ontology analysis revealed that blood vessel development, regulation of cell proliferation, cell motion, regulation of apoptosis/cell death and angiogenesis were among the most enriched gene ontology terms for YY1 positively-regulated genes (Fig. 5C), whereas cell adhesion, neuron development, transmembrane receptor protein tyrosine kinase signaling pathway, intracellular signaling cascade and cell projection organization were among the most enriched gene ontology terms for YY1 negatively-regulated genes (Supplementary Fig. 4).

As regulation of cell proliferation was one of the well-characterized functions of YY1, we focused on studying the involvement of SET7/9-mediated YY1 methylation in this aspect of YY1 function. HeLa cells were transfected with control siRNA or siRNA specifically targeting *YY1* or *SET7/9* followed by RT-qPCR analysis to examine the expression of selected YY1 positively-regulated genes based on Gro-seq, which have been shown to involve in cell cycle regulation (Fig. 5D,E). It was found that the expression of these genes was decreased to a similar extent when knocking down of YY1 or SET7/9 (Fig. 5E).

To further test whether SET7/9-mediated YY1 methylation is involved in YY1-regulated gene transcriptional program, HeLa cells were transfected with control vector or vectors expressing YY1(wt), YY1(K173R) or YY1(K411R), followed by RT-qPCR analysis to examine the expression of several genes mentioned above. Unexpectedly, instead of further activating them, over-expression of YY1 resulted in either a decreased or unaltered expression of these genes (Fig. 5F and data not shown), which was consistent with previous reports that YY1 as well as its human homolog, YY2, could be switched from an activator to repressor when present at high levels^47^. Nevertheless, YY1’s repressive effects on these genes appeared to be attenuated when K173 or K411 was mutated (Fig. 5F). To further demonstrate YY1’s repressive effects on gene transcription when present at high levels were dependent on K173 or K411, HeLa cells were transfected with luciferase reporter vector containing YY1 consensus binding site (pGL2-YY1(wt)-*luc*), and vectors expressing YY1(wt), YY1(K173R) or YY1(K411R), followed by luciferase reporter activity measurement. Consistently, over-expression of YY1(wt) led to a significant decrease of luciferase reporter activity, whereas YY1(K173R) and YY1(K411R) did so in a less extent (Supplementary Fig. 5). Taken together, these data were consistent with our observation that K173 and K411 were involved in YY1 DNA-binding activity, suggesting SET7/9-mediated YY1 methylation might involve in YY1-regulated gene transcription.

### SET7/9-mediated YY1 methylation is involved in YY1-regulated cell proliferation

**Figure 6:**
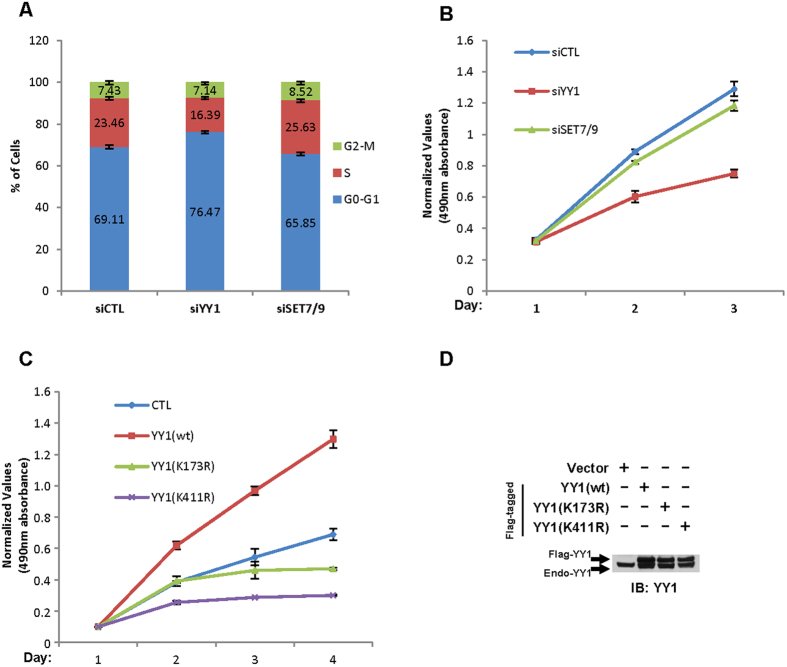
SET7/9-mediated YY1 methylation is involved in YY1-regulated cell proliferation. (**A**) HeLa cells were transfected with control siRNA or siRNA specifically targeting *YY1* or *SET7/9*, followed by flow cytometry analysis. Percentage of cells in each cell cycle phase, G0-G1, S and G2-M, was shown as indicated (±s.e.m.). The change of percentage of cells in G0-G1 and S phases between siCTL and siYY1 were both significant (P < 0.001). Experiments were repeated three times and representative data was shown. (**B**) HeLa cells were transfected with control siRNA or siRNA specifically targeting *YY1* or *SET7/9*, followed by MTS assay to measure cell proliferation rate for two consecutive days. The change of absorbance between siCTL and siYY1 in both day 2 and 3 were significant (P < 0.01 and P < 0.001, respectively). Experiments were repeated three times and representative data was shown. (**C**) HeLa cells stably expressing control vector, Flag-tagged YY1(wt), YY1(K173R) or YY1(K411R) were seeded at the same density and their proliferation rate was monitored for three consecutive days by MTS assay. Significant test was performed for the change of absorbance between different conditions. In day 2: CTL vs YY1(wt) (P < 0.001), CTL vs YY1(K411R) (P < 0.001); In day 3: CTL vs YY1(wt) (P < 0.001), CTL vs YY1(K411R) (P < 0.01); In day 4: CTL vs YY1(wt) (P < 0.001), CTL vs YY1(K173R) (P < 0.01), CTL vs YY1(K411R) (P < 0.01). Experiments were repeated three times and representative data was shown. (**D**) The levels of stably expressed Flag-tagged YY1(wt), YY1(K173R) and YY1(K411R) as described in (**C**) was examined through immunoblotting using anti-YY1 antibody. Endo-YY1: endogenous YY1.

It has been reported previously that MEF cells from mice carrying *yy1* alleles expressing various amounts of YY1 display a dosage-dependent requirement of YY1 for cell proliferation. Accordingly, inhibition of YY1 in cultured cells leads to cytokinesis defects and cell cycle arrest^11^. YY1 function in cell proliferation is mainly due to its critical role in regulating gene transcription. Our Gro-seq analysis revealed that a substantial list of genes with implications in cell proliferation was indeed regulated by YY1. To examine whether SET7/9-mediated YY1 methylation is involved in YY1-regulated cell proliferation, firstly, HeLa cells were transfected with control siRNA or siRNA specifically targeting *YY1* or *SET7/9* followed by cell cycle analysis using flow cytometry and cell viability measurement using MTS assay (Fig. 6A,B). As reported previously, knock-down of YY1 caused a significant increase of the number of cells to arrest in G1 phase (Fig. 6A)^11^. Consistently, knock-down of YY1 led to a decreased cell proliferation rate measured by MTS assay (Fig. 6B). To our surprise, SET7/9 displayed no significant effect, which might be due to the fact that SET7/9 has a plethora of substrates *in vivo* and its impact on cell cycle progression and cell proliferation is context specific (Fig. 6A,B)^36^,^37^. Secondly, HeLa cells stably expressing a control vector or vectors expressing YY1(wt), YY1(K173R) or YY1(K411R) were plated at the same density, and their proliferation rate was recorded through MTS assay. In accordance with its role in promoting cell proliferation, cells stably expressing YY1(wt) grew much faster compared to control cells, whereas those expressing YY1(K411R) grew slower than control cells starting from the next day after seeding, and those expressing YY1(K173R) also displayed retarded cell growth two days after, exhibiting a dominant-negative effect (Fig. 6C). The levels of stably expressed YY1(wt), YY1(K173R) and YY1(K411R) was comparable examined through immunoblotting (Fig. 6D).

## Discussion

YY1 has been shown to be a target of many types of post-translational modifications, including poly(ADP-ribosyl)ation, ubiquitination, acetylation, O-linked glycosylation, S-nitrosation, sumoylation, and phosphorylation. Despite it has been found to interact with several histone methyltransferases, such as PRMT1, EZH1 and EZH2, whether YY1 is targeted by methylation remains unknown. In the present study, we provided evidence that YY1 was methylated by SET7/9 at two lysine residues, which were shown to be important for its DNA-binding activity both *in vitro* and in cultured cells. Importantly, SET7/9-mediated YY1 methylation was shown to involve in YY1-regulated gene transcription and cell proliferation, linking YY1 methylation with its biological functions for the first time.

Through screening a panel of histone lysine methyltransferases, it was found that YY1 was robustly methylated by SET7/9 *in vitro* (Fig. 1A,B). As some of the enzymes could work under different conditions and many of the known lysine methyltransferases were not included in our screening, it remains unknown whether YY1 is targeted for methylation by other enzymes. Further mutagenesis analysis revealed that YY1 was targeted at two lysine residues by SET7/9 (Fig. 1C–F). LSD1, the demethylase with opposing activity to SET7/9 on histone as well as several non-histone proteins, displayed no demethylase activity towards SET7/9-mediated YY1 methylation (Fig. 2D and Supplementary Fig. 2A). Similarly, it has been reported previously that lysine methylation on p53 protein mediated by SET7/9 was not a target of LSD1^48^. Therefore, hunting for demethylases with opposite activity to SET7/9 in addition to LSD1 is of great interest for future investigation.

The high degree of conservation of both K173 and K411 in YY1 protein pointed to the functional importance of the methylation found on these two lysines (Supplementary Fig. 1G and H). As YY1 exerts its biological functions primarily through its association with chromatin DNA, we therefore tested whether SET7/9-mediated YY1 methylation regulates its DNA-binding activity. Interestingly, substitution of lysine 173 or 411 to arginine (K173R or K411R) attenuated YY1 binding affinity with its consensus DNA binding motif (Fig. 3A–D). The weaker binding of YY1(K411R) with DNA compared to YY1(wt) was most likely due to that K411 is in close proximity to zinc finger domains, mutation of which interfered zinc fingers’ binding to DNA, whereas the weaker binding of YY1(K173R) with DNA compared to YY1(wt) might be linked to the cross-talk between YY1K173me1 and other post-translational modifications (PTMs) located at the central glycine-rich region, such as acetylation and phosphorylation^23^,^30^. In addition, the glycine-rich region can function as a protein-protein interaction domain to interact with several proteins, including p300, HDACs, c-Myc, p53 and p14^ARF(3)^. We therefore speculate that K173 methylation might be recognized by a “reader” protein, which is capable of regulating YY1 DNA-binding activity.

Our observation that SET7/9-mediated YY1 methylation affected its DNA-binding activity *in vitro* prompted us to examine whether its binding to chromatin in cultured cells was also regulated by SET7/9. To our surprise, SET7/9-regulated YY1 binding seemed to be gene specific. We speculate that co-localization of SET7/9 might be one of the determinants for YY1 chromatin targeting to specific genes, similar as other chromatin modifying enzymes^3^. Unfortunately, commercially available anti-SET7/9 antibodies limited us from a successful ChIP-seq for SET7/9. YY1 has been shown to be targeted for post-translational modifications by several enzymes, such as PLK1, Aurora B, p300, CBP, PCAF, HDAC1, HDAC2 and HDAC3, with many of them affecting YY1 DNA-binding activity *in vitro* and/or chromatin targeting in cultured cells. Therefore, one can envision that, through association with different enzymes, YY1 achieves its gene specific targeting and hence activates or represses gene transcription. Indeed, our Gro-seq analysis revealed that YY1 regulated the expression of a substantial list of genes (Fig. 5B). Careful examination of the promoter regions of these genes revealed that majority of them exhibited no YY1 binding, suggesting that they might be regulated in a distal fashion. Moreover, the nearest YY1 binding site away from these promoters often located on the promoter region of another gene, transcription of which was not affected by YY1 or SET7/9, suggesting a promoter to promoter communication in YY1-mediated gene transcriptional regulation. Indeed, genome-wide study suggested a wide spreading promoter-centered chromatin interactions dictated for transcription regulation^49^,^50^.

As one of the well-studied functions of YY1 is regulation of cell proliferation, we tested whether SET7/9-mediated YY1 methylation involved in such process. Interestingly, substitution of K173 or K411 to arginine completely abolished the effects of YY1 on promoting cell proliferation (Fig. 6C). Ample studies have shown an intimate link between YY1 function in cell proliferation and tumorigenesis. Therefore, it will be valuable to investigate whether YY1 methylation is enriched and therefore serves as a biomarker in cancers.

## Materials and Methods

### Plasmids and Cloning Procedures

YY1, SET7/9, G9a, MLL1, SET1A, SET1B, SUV4-20H1, SUV4-20H2, ESET, SUV39H1, SUV39H2, DOT1L, SET8, RIZ1, EZH2 full-length (FL) or truncations were PCR-amplified from cDNA samples prepared from HEK293T cells by using KOD Hot Start DNA Polymerase (Novagen) and then cloned into p3XFLAG-CMV-10 (Sigma), pcDNA3-HA (Invitrogen), pEGFP-C3 (Clontech), pET-28a (+) (Novagen) or pGEX-6P-1 (Promega) expression vectors. All point mutations were generated by using QuikChange Lightning Site-Directed Mutagenesis Kit (Stratagene). Luciferase reporter constructs containing YY1 consensus binding motif (CGCTCCCCGGCCATCTTGGCGGCT GGT) or its mutant form (CGCTCCGCGATTATCTTGGCGGCTGGT) were made in pGL2 vector (Promega).

### siRNAs, Antibodies, Peptides and Proteins

siRNA specifically targeting *YY1* (GACGACGACTACATTGAACAA), *SET7/9* (TAGGGCCAGGGTATTATTATA) or *LSD1*/*AOF2* (CTGGAAATGACTATGATTTAA) was purchased from Qiagen. Anti-YY1 K173me1 and anti-YY1 K411me1 antibodies were generated by GenScript, Inc. Antigen (peptide sequence) used for generating anti-YY1 K173me1 and anti-YY1 K411me1 was CSGGGRVK(me1)KGGGKKS and CKSHILTHAKAK(me1)NNQ, respectively; anti-Flag (F1804) antibody was purchased from Sigma; anti-SET7/9 (07–314) was purchased from Upstate; anti-LSD1/AOF2 (A300-215A) was purchased from Bethyl Laboratory, Inc; anti-YY1(H-10) (SC-7341) and anti-GAPDH (SC-25778) was purchased from Santa Cruz Biotechnology. Flag peptide (F-4799) was purchased from Sigma; YY1K173me1 and YY1K411me1 peptides shared the same sequence as the ones used for antibody production.

### Plasmids or siRNA Transfection, RNA Isolation, and RT-qPCR

Plasmid and siRNA transfection was performed using Lipofectamine 2000 (Invitrogen) according to the manufacturer’s protocol. Total RNA was isolated using RNeasy Mini Kit (Qiagen) following the manufacturer’s protocol. First-strand cDNA synthesis from total RNA was carried out using iScript™ cDNA Synthesis Kit (Bio-Rad), followed by quantitative PCR (qPCR) using Mx3005 machine (Stratagene). All RT-qPCRs were repeated at least three times and representative results were shown. Sequence information for all primers used to check gene expression was presented in Supplementary Table 2.

### Protein Immunoblotting and Immunoprecipitation

Protein immunoblotting and immunoprecipitation were performed following the protocol described previously^51^,^52^.

### Purification of Bacterially-expressed Proteins

His-tagged YY1 full-length (FL), truncations, point mutations were expressed in BL21 (DE3) bacterial cells (Stratagene) and purified by using Ni-NTA agarose (Qiagen), following the protocol described previously^51^,^52^.

### *In vitro* Methylation Assay

*In vitro* methylation assay was performed by mixing purified bacterially-expressed YY1 full length (FL), truncations, point mutations or core histones with histone lysine methyltransferases, either purified from bacterial cells or HEK293T cells with over-expression, in methylation buffer (50 mM Tris-HCl, pH 8.0, 20 mM KCl, 5 mM DTT, 4 mM EDTA) in the presence of 2 μCi L-[*methyl*-^3^H]-methionine at 37 °C for 1 hr. The reaction was stopped by adding SDS sample buffer followed by SDS-PAGE gel and autoradiogram. For *in vitro* methylation assay using peptides as substrates, enzyme (SET7/9) was removed by adding Ni-NTA agarose before dot blot assay.

### Generation of SET7/9 Knock-out Cell Lines Using CRISPR/Cas9 Gene Editing Technology

SET7/9 knockout (KO) HeLa cells were generated using CRISPR/Cas9 system as described previously^53^. gRNA targeting sequence (5′-TAGCGACGACGAGATGGTGG-3′) was cloned into gRNA cloning vector (Addgene, 41824) and confirmed by sequencing. To screen for SET7/9 KO clones, HeLa cells were transfected with pcDNA3.3-hCas9 and gRNA expression vectors (Addgene 41815), followed by G418 selection (0.5 mg/ml). Single colonies were subjected to immunoblotting (IB) using anti-SET7/9 antibody to select knock-out ones, which were further validated by PCR using genomic DNA as template followed by Sanger sequencing. The sequencing information for primer sets used was as follows: Forward (F) 5′-CTCCTCCTCCTCCAAACTCG-3′; Reverse (R) 5′-ACTCCTTCCGCGCTCCAG-3′.

### Electrophoretic Mobility Shift Assay (EMSA)

YY1 DNA-binding activity *in vitro* was examined through EMSA by using the LightShift Chemiluminescent EMSA Kit (20148) from Pierce following manufacturer’s protocol. Briefly, whole cell lysates were prepared in lysis buffer containing 20 mM HEPES, pH 8.0, 1.5 mM MgCl_2_, 25% glycerol, 420 mM NaCl, 1 mM DTT, 0.4 mM EDTA, followed by repeated freeze-and-thaw cycles and sonication. Each EMSA reaction was set up at a final volume of 20 μl. Around 15 to 30 μg whole cell lysates were mixed with 10 X binding buffer (100 mM Tris, 500 mM KCl, 10 mM DTT; pH 7.5) (2 μl), 50% glycerol (1 μl), 100 mM MgCl_2_ (1 μl), 1 mg/ml Poly (dI.dC) (1 μl), 1% NP-40 (1 μl) in the presence or absence of unlabeled YY1 consensus binding motif (2 pmol/ μl) (2 μl)(forward: 5′-CGCTCCCCGGCCATCTTGGCGGCTGGT-3′; reverse: 5′- ACCAGCCGCCAAGATGGCCGGGGAGCG-3′) or its mutant form (2 pmol/ μl) (2 μl) (forward: 5′-CGCTCCGCGATTATCTTGGCGGCTGGT-3′; reverse: 5′- ACCAGCCGCCAAGATAATCGCGGAGCG-3′) on ice for 10 mins, followed by incubation at room temperature for 20 mins. Finally, biotin-labeled YY1 consensus binding motif (10 fmol/ μl) (2 μl) (forward: 5′biotin-CGCTCCCCGGCCATCTTGGCGGCTGGT-3′; reverse: 5′biotin-ACCAGCCGCCAAGATGGCCGGGGAGCG-3′) or its mutant form (2 pmol/ μl) (2 μl) (forward: 5′biotin-CGCTCCGCGATTATCTTGGCGGCTGGT-3′; reverse: 5′ biotin-ACCAGCCGCCAAGATAATCGCGGAGCG-3′) was added and incubated at room temperature for 10 mins before adding 5X loading buffer. The protein-DNA complex was resolved by 6% DNA retardation gel (Life Technology, EC63652BOX), followed by electrophoretic transfer to Nylon membrane (Pierce, 77016). The membrane was then cross-linked and subjected to detection using the chemiluminescent nucleic acid detection module (Part No. 89880) provided in the kit following manufacturer’s protocol.

### Chromatin Immunoprecipitation (ChIP)

ChIP was performed following the protocol described previously^51^,^52^. Briefly, cells were fixed with 1% formaldehyde (Sigma) for 10 mins at room temperature (RT). Fixation was stopped by adding glycine (0.125 M) and incubating for 5 min at room temperature (RT), followed by washing with PBS buffer twice. Chromatin DNA was sheared to 300~500 bp average in size through sonication. Resultant was immunoprecipitated with control IgG or specific antibodies overnight at 4 °C, followed by incubation with protein G magnetic beads (Invitrogen) for an additional 2 hrs. After washing and elution, the protein-DNA complex was reversed by heating at 65 °C overnight. Immunoprecipitated DNA was purified by using QIAquick spin columns (Qiagen) and analyzed by q-PCR using Mx3005 machine (Stratagene). All ChIP-qPCRs were repeated at least three times and representative results were shown. Sequence information for all primers used for ChIP was presented in Supplementary Table 2.

### Chromatin Immunoprecipitation Coupled with High Throughput Sequencing (ChIP-Seq)

ChIP-seq sample preparation and computational analysis of ChIP-seq data were performed as described previously with minor modifications^51^. Library construction: the libraries were constructed following Illumina ChIP-seq Sample prep kit. Briefly, ChIP DNA was end-blunted and added with an ‘A’ base so the adaptors from Illumina with a ‘T’ can ligate on the ends. Then 200–400 bp fragments are gel-isolated and purified. The library was amplified by 18 cycles of PCR. Primary analysis of ChIP-Seq datasets: the image analysis and base calling were performed by using Illumina’s Genome Analysis pipeline. The sequencing reads were aligned to the human genome UCSC build hg18 by using Bowtie2 alignment program. Only uniquely aligned reads were kept. The aligned reads were used for peak finding with HOMER (http://biowhat.ucsd.edu/homer). Of note, promoter (TSS)-associated peaks were defined as those with peak center falling between 100 bp downstream of and 5,000 bp upstream of TSS region. Similarly, enriched motifs in identified peaks were searched using HOMER. YY1 ChIP-seq data were deposited in the Gene Expression Omnibus database under accession GSE69759.

### Identification of Proteins Differentially Associated with YY1(wt), YY1(K173R) and YY1(K411R)

To identify proteins differentially associated with YY1(wt), YY1(K173R) and YY1(K411R), whole cell lysates were prepared in lysis buffer containing 20 mM HEPES pH 8.0, 1.5 mM MgCl_2_, 150 mM NaCl, 25% glycerol, 1 mM DTT, 0.2 mM EDTA, 1% Triton X-100 from HeLa cells stably expressing p3XFLAG-CMV-10-YY1(wt), YY1(K173R) or YY1(K411R), and then subjected to affinity purification by using anti-Flag M2-agarose (Sigma, A2220), washed extensively and eluted with 3XFlag peptides. Elutes were then separated by SDS-PAGE gel followed by silver staining using the SilverQuest Silver Staining Kit (Life Technology, LC6070) following the manufacturer’s protocol. Differentially present bands were subjected to in gel digestion and tandem mass spectroscopy (LC-MS/MS) analysis following the protocol as previously described^51^.

### Global Run-On Coupled to High-Throughput Sequencing (Gro-seq)

Gro-seq was performed following the protocol as described previously^51^.

### Gro-Seq Analysis

The sequencing reads were aligned to hg18 Refseq database by using Bowtie2. EdgeR (http://www.bioconductor.org/packages/release/bioc/html/edgeR.html) was then used to compute the significance of the differential gene expression (P < 0.001). The common artifacts derived from clonal amplification were circumvented by considering maximal three tags from each unique genomic position. Gene ontology analysis for genes regulated by YY1 was done using David^54^. All Gro-seq data were deposited in the Gene Expression Omnibus database under accession GSE69759.

### Luciferase Reporter Assay

HeLa cells were transfected with Renilla luciferase reporter vector (Promega) and luciferase reporter vectors containing YY1 consensus binding site (pGL2-YY1(wt)-*luc*) or its mutant form (pGL2-YY1(m)-*luc*) in the presence or absence of YY1(wt), YY1(K173R) or YY1(K411R) for 48 hrs. Cells were then washed with PBS buffer twice and lysed with 1X passive lysis buffer (Promega) before measurement of luciferase reporter activity by using the Dual-Luciferase Reporter Assay System (Promega).

### Flow cytometry

Flow cytometry was performed as previously reported^52^. HeLa cells were trypsinized, washed with PBS and fixed with ethanol at 4 °C overnight. Cells were then washed with PBS and stained with PI/Triton X-100 staining solution (0.1% (v/v) Triton X-100, 0.2 mg/ml DNase-free RNase A (Sigma), 0.02 mg/ml propidium iodide (Roche)) at 37 °C for 15 min. DNA content was then measured and about 10^5^ events were analysed for each sample. Data were analysed using CellQuest (Becton Dickinson) and ModFit LT (Verity Software House).

### Cell Proliferation Assay

Cell proliferation assay was performed as previously reported^52^. Cell viability was measured by using a CellTiter 96 AQueous one solution cell proliferation assay kit (Promega) following the manufacturer’s protocol. Briefly, HeLa cells stably expressing control vector, YY1(wt), YY1(K173R) or YY1(K411R) were seeded at the same density and their proliferation rate was monitored for consecutive two days. Specifically, 20 μl of CellTiter 96 AQueous one solution reagent was added per 100 μl of culture medium, and the culture plates were incubated for 1 h at 37 °C in a humidified, 5% CO_2_ atmosphere incubator. The reaction was stopped by adding 25 μl of 10% SDS. Data was recorded at wavelength 490 nm using a Thermo Multiskan MK3 Microplate Reader.

## Additional Information

**How to cite this article**: Zhang, W.- *et al.* Regulation of Transcription Factor Yin Yang 1 by SET7/9-mediated Lysine Methylation. *Sci. Rep.* **6**, 21718; doi: 10.1038/srep21718 (2016).

## Supplementary Material

Supplementary Information
