# Supplementary material for: Regulation of Transcription Factor Yin Yang 1 by SET7/9-mediated Lysine Methylation
Source: Sci Rep. 2016 Feb 23;6:21718. doi: 10.1038/srep21718 (PMC4763200; doi:10.1038/srep21718)
Supplement: Supplementary Information [file srep21718-s1.pdf]

Regulation of Transcription Factor Yin Yang 1 by SET7/9-mediated Lysine Methylation

Wen-juan Zhang, Xiao-nan Wu, Tao-tao Shi, Huan-teng Xu, Jia Yi, Hai-feng Shen, Ming-feng Huang, Xing-yi Shu, Fei-fei Wang, Bing-ling Peng, Rong-quan Xiao, Wei-wei Gao, Jian-cheng Ding, Wen Liu

Supplementary Information

Supplementary Figure Legends

Figure S1. SET7/9 methylates YY1 *in vitro*.

(A) *In vitro* methylation assay was performed by mixing all enzymes shown in Fig. 1A and core histones, followed by autoradiogram. Of note, G9a and SET7/9 were active under current conditions tested.

(B) The expression of all enzymes included in Fig. 1A was shown by coomassie blue staining (C.B.S).

(C) Long exposure of the films shown in Fig. 1B. White arrows indicate automethylation (auto-me) of KMTs; Black arrows indicate methylation of YY1 (YY1(me)).

(D) *In vitro* methylation assay was performed by mixing all enzymes shown in Fig. 1B and core histones, followed by autoradiogram.

(E) The expression of all enzymes included in Fig. 1B was shown by immunoblotting with antibodies as indicated.

(F) Human YY1 sequence. Amino acids were color coded identical as shown in Fig. 1C. Lysine residues embedded in a potential SET7/9 methylation site were underlined， which were substituted to arginine as shown in Fig. 1E and 1F.

(G, H) Sequence alignment of the region surrounding the two lysine residues, 173 (G) and 411 (H), methylated by SET7/9 (boxed) in paralogous YY1 genes in various organisms by using Clustal Omega. Asterisk (*) indicates positions which have fully conserved residue; Colon (:) indicates conservation between groups of strongly similar properties; Period (.) indicates

conservation between groups of weakly similar properties. *Hs, Homo sapiens*; *Mm, Mus musculus*; *Rn, Rattus norvegicus*; *Gg, Gallus gallus*; *Bt, Bos Taurus*; *Xl, Xenopus laevis*; *Xt, Xenopus (Silurana) tropicalis*; *Dr, Danio rerio*.

Figure S2. Effects of knock-down of SET7/9 or LSD1 on YY1 K173 and K411 methylation.

(A) HeLa cells transfected with control siRNA or siRNA specifically targeting *SET7/9* or *LSD1* were subjected to IB with antibodies as indicated.

(B) Genomic DNA was extracted from SET7/9 knock-out (KO) cells generated by CRISPR/Cas9 system, followed by PCR using specific primer sets surrounding gRNA targeting region (boxed in blue). The resultant PCR products were subjected to Sanger sequencing and genomic region with deletions was shown, with 20 basepair (bp) removed as shown in red. Translation start codon (ATG) (dark red);

Figure S3. SET7/9-mediated YY1 methylation regulates YY1 association with chromatin.

(A) HeLa cells were transfected with control vector or vectors expressing SET7/9, followed by ChIP with anti-YY1 antibody and q-PCR with primers specifically targeting promoter regions of selected genes as indicated. ChIP signals were presented as percentage of inputs (± s.e.m., $^{**}P<0.01$, $^{***}P<0.001$). Experiments were repeated three times and representative data was shown.

(B, C) HeLa cells were transfected with control siRNA or siRNA specifically targeting *SET7/9*, followed by ChIP with anti-YY1 antibody and q-PCR with primers specifically targeting promoter regions of selected genes as indicated. Both promoters with (B) and without (C) altered YY1 binding upon SET7/9 knock-down were shown. ChIP signals were presented as percentage of inputs (± s.e.m., $^{*}P<0.05$, $^{**}P<0.01$, $^{***}P<0.001$, N.S: not significant). Experiments were repeated three times and representative data was shown.

(D) HeLa cells were transfected with vectors expressing Flag-tagged YY1(wt), YY1(K173R) or YY1(K411R), followed by ChIP with anti-Flag antibody and q-PCR with primers specifically targeting promoter regions of selected genes as indicated. ChIP signals were presented as percentage of inputs (± s.e.m., $^{**}P<0.01$, $^{***}P<0.001$). Experiments were repeated three times and representative data was shown.

(E) HeLa cells stably expressing Flag-tagged YY1(wt) or YY1(K411R) were subjected to affinity purification with Flag M2 agarose. The resultant proteins were separated by SDS-PAGE gel, followed by silver staining. Four bands (1-4) specifically present in YY1(wt) sample were cut and subjected to mass spectrometry (MS) analysis.

Figure S4. Gene ontology analysis for genes negatively-regulated by YY1 identified through Gro-seq. Gene ontology analysis was performed for genes negatively-regulated by YY1 as shown in Fig. 5B using DAVID. Top ten enriched gene ontology (GO) terms were shown.

Figure S5. K173 and K411 were involved in YY1-regulated gene transcription. HeLa cells were transfected with luciferase reporter containing YY1 consensus binding site (pGL2-YY1(wt)-*luc*) in the presence or absence of control vector or vectors expressing YY1(wt), YY1(K173R) or YY1(K411R), followed by luciferase reporter activity measurement (± s.e.m., $^{*}P<0.05$, $^{**}P<0.01$, $^{***}P<0.001$). Experiments were repeated three times and representative data was shown.

Supplementary Table Legends

Table S1. Mass spectrometry (MS) analysis of proteins specifically present in YY1 interactome. Bands (1-4) specifically present in YY1(wt) sample as shown in Fig. 4F were cut and subjected to mass spectrometry (MS) analysis.

Table S2. Sequence information for all qPCR primers used in the current study. Sequence information of qPCR primers specifically targeting to pGL2-*luc* vector, *p53*, *RAD1*, *ABL1*, *CCND1*, *CCNT2*, *CCNA2*, *SDF4*, *PAGR1*, *CDK5RAP2* or *SLX4IP* gene promoter regions to examine YY1 binding after ChIP, and primers specifically targeting to *CDK6*, *KIF18A*, *RB1*, *BCAT1*, *CDCA2*, *PLAGL1*, *NOTCH2*, *ASPM*, *CXCL16*, or *E2F7* to examine their expression levels were shown. F: forward; R: reverse.

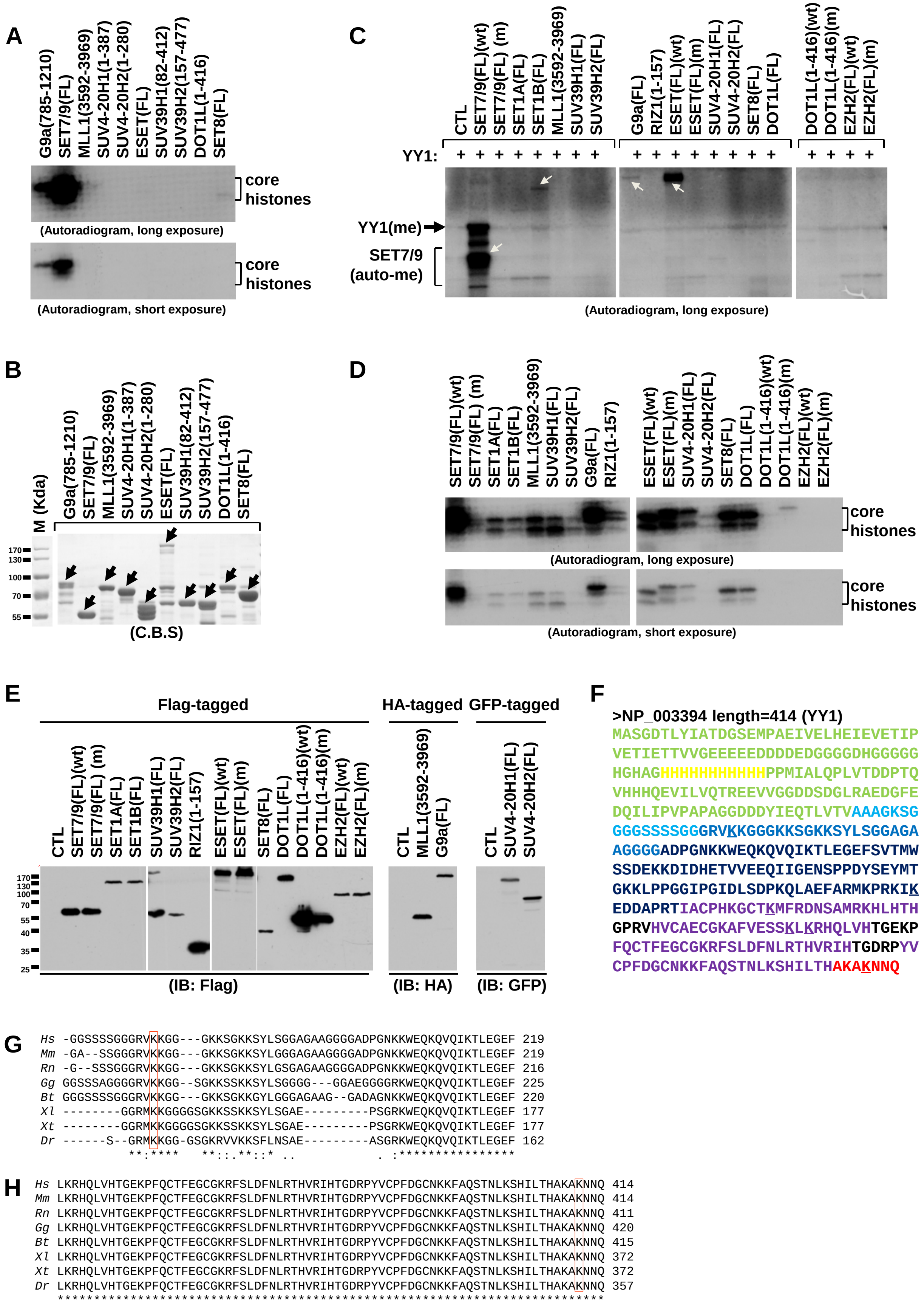
A
G9a(785-1210)
SET7/9(FL)
MLL1(3592-3969)
SUV4-20H1(1-387)
SUV4-20H2(1-280)
ESET(FL)
SUV39H1(82-412)
SUV39H2(157-477)
DOT1L(1-416)
SET8(FL)
core histones
(Autoradiogram, long exposure)
core histones
(Autoradiogram, short exposure)
B
M (Kda)
170
130
100
70
55
(C.B.S)
C
CTL
SET7/9(FL)(wt)
SET7/9(FL) (m)
SET1A(FL)
SET1B(FL)
MLL1(3592-3969)
SUV39H1(FL)
SUV39H2(FL)
G9a(FL)
RIZ1(1-157)
ESET(FL)(wt)
ESET(FL)(m)
SUV4-20H1(FL)
SUV4-20H2(FL)
SET8(FL)
DOT1L(FL)
DOT1L(1-416)(wt)
DOT1L(1-416)(m)
EZH2(FL)(wt)
EZH2(FL)(m)
YY1:
YY1(me)
SET7/9 (auto-me)
(Autoradiogram, long exposure)
D
core histones
(Autoradiogram, long exposure)
core histones
(Autoradiogram, short exposure)
E
Flag-tagged
HA-tagged
GFP-tagged
170
130
100
70
55
40
35
25
(IB: Flag)
(IB: HA)
(IB: GFP)
F
>NP_003394 length=414 (YY1)
MASGDTLYIATDGSEMPAEIVELHEIEVETIP
VETIETTVVGEEEEEDDDDEDGGGGDHGGGGG
HGHAGHHHHHHHHHHHPPMIALQPLVTDDPTQ
VHHHQEVILVQTREEVVGGDDSDGLRAEDGFE
DQILIPVPAPAGGDDDYIEQTLVTVAAAGKSG
GGGSSSSGGGRVKKGGGKKSGKKSYLSGGAGA
AGGGGADPGNKKWEQKQVQIKTLEGEFSVTMW
SSDEKKDIDHETVVEEQIIGENSPPDYSEYMT
GKKLPPGGIPGIDLSDPKQLAEFARMKPRKIK
EDDAPRTIACPHKGCTKMFRDNSAMRKHLHTH
GPRVHVCAECGKAFVESSKLKRHQLVHTGEKP
FQCTFEGCGKRFSLDFNLRTHVRIHTGDRPYV
CPFDGCNKKFAQSTNLKSHILTHAKAKNNQ
G
Hs -GGSSSSGGGRVKKGG---GKKSGKKSYLSGGAGAAGGGGADPGNKKWEQKQVQIKTLEGEF 219
Mm -GA--SSGGGRVKKGG---GKKSGKKSYLGGGAGAAGGGGADPGNKKWEQKQVQIKTLEGEF 219
Rn -G--SSSGGGRVKKGG---GKKSGKKSYLSGSAGAAGGGGADPGNKKWEQKQVQIKTLEGEF 216
Gg GGSSSAGGGGRVKKGG--SGKKSSKKSYLSGGGG---GGAEGGGGRKWEQKQVQIKTLEGEF 225
Bt GGGSSSSGGGRVKKGG---GKKSGKKGYLGGGAGAAG--GADAGNKKWEQKQVQIKTLEGEF 220
Xl --------GGRMKKGGGGSGKKSSKKSYLSGAE---------PSGRKWEQKQVQIKTLEGEF 177
Xt --------GGRMKKGGGGSGKKSSKKSYLSGAE---------PSGRKWEQKQVQIKTLEGEF 177
Dr ------S--GRMKKGG-GSGKRVVKKSFLNSAE---------ASGRKWEQKQVQIKTLEGEF 162
H
Hs LKRHQLVHTGEKPFQCTFEGCGKRFSLDFNLRTHVRIHTGDRPYVCPFDGCNKKFAQSTNLKSHILTHAKAKNNQ 414
Mm LKRHQLVHTGEKPFQCTFEGCGKRFSLDFNLRTHVRIHTGDRPYVCPFDGCNKKFAQSTNLKSHILTHAKAKNNQ 414
Rn LKRHQLVHTGEKPFQCTFEGCGKRFSLDFNLRTHVRIHTGDRPYVCPFDGCNKKFAQSTNLKSHILTHAKAKNNQ 411
Gg LKRHQLVHTGEKPFQCTFEGCGKRFSLDFNLRTHVRIHTGDRPYVCPFDGCNKKFAQSTNLKSHILTHAKAKNNQ 420
Bt LKRHQLVHTGEKPFQCTFEGCGKRFSLDFNLRTHVRIHTGDRPYVCPFDGCNKKFAQSTNLKSHILTHAKAKNNQ 415
Xl LKRHQLVHTGEKPFQCTFEGCGKRFSLDFNLRTHVRIHTGDRPYVCPFDGCNKKFAQSTNLKSHILTHAKAKNNQ 372
Xt LKRHQLVHTGEKPFQCTFEGCGKRFSLDFNLRTHVRIHTGDRPYVCPFDGCNKKFAQSTNLKSHILTHAKAKNNQ 372
Dr LKRHQLVHTGEKPFQCTFEGCGKRFSLDFNLRTHVRIHTGDRPYVCPFDGCNKKFAQSTNLKSHILTHAKAKNNQ 357

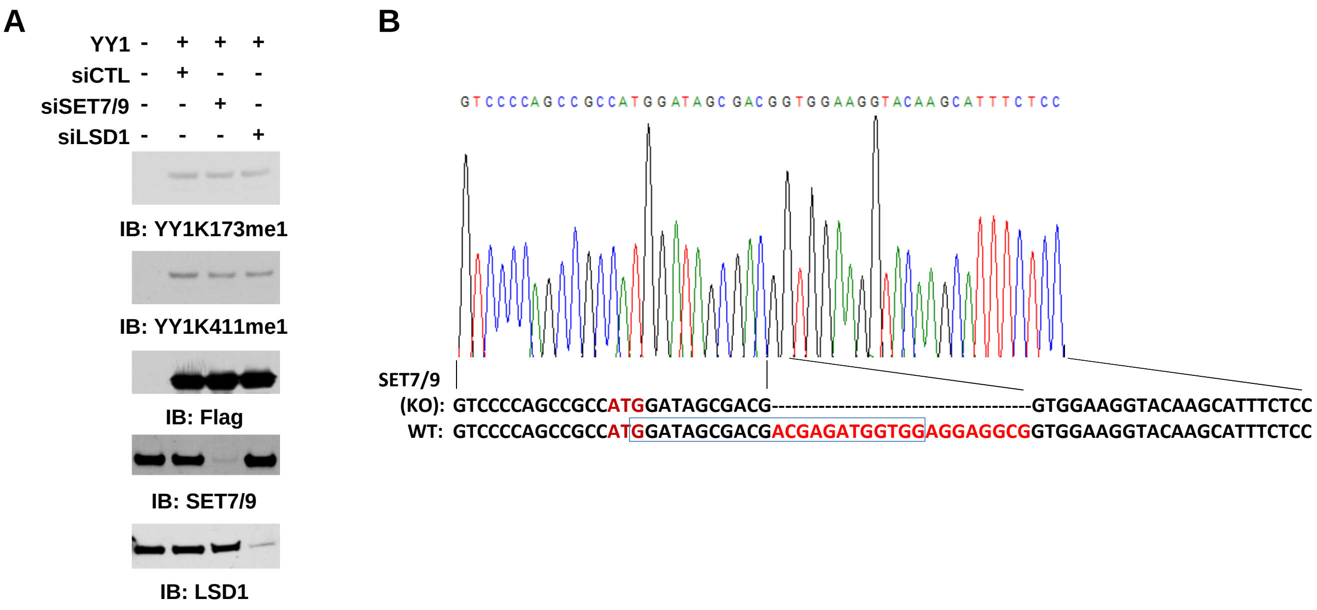

A
YY1 - + + +
siCTL - + - -
siSET7/9 - - + -
siLSD1 - - - +
IB: YY1K173me1
IB: YY1K411me1
IB: Flag
IB: SET7/9
IB: LSD1
B
GTCCCCAGCCGCCATGGATAGCGACGGTGGAAGGTACAAGCATTTCTCC
SET7/9
(KO): GTCCCCAGCCGCCATGGATAGCGACG------------------------------------GTGGAAGGTACAAGCATTTCTCC
WT: GTCCCCAGCCGCCATGGATAGCGACGACGAGATGGTGGAGGAGGCGGTGGAAGGTACAAGCATTTCTCC

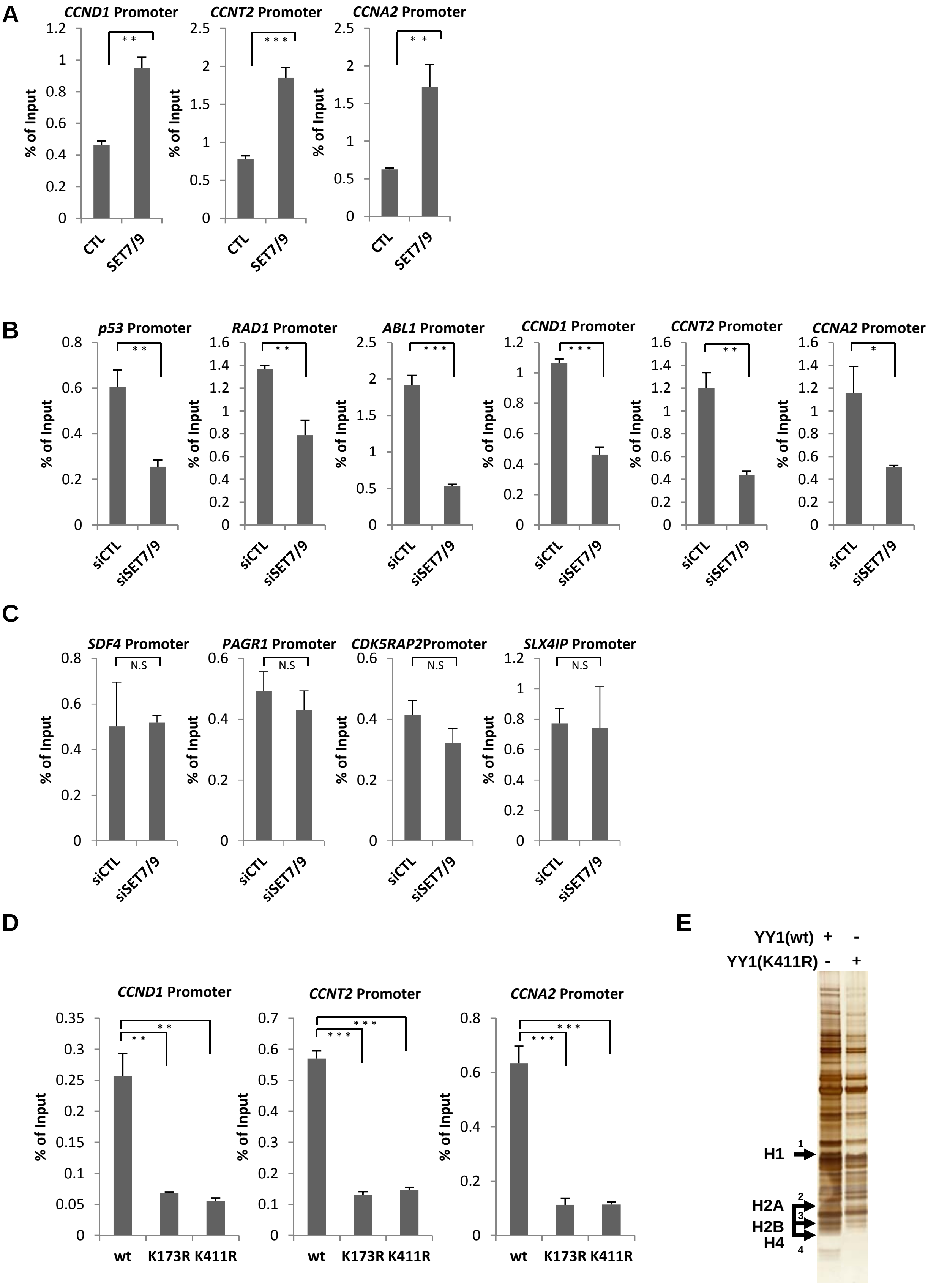
A
CCND1 Promoter
CCNT2 Promoter
CCNA2 Promoter
% of Input
CTL
SET7/9
B
p53 Promoter
RAD1 Promoter
ABL1 Promoter
CCND1 Promoter
CCNT2 Promoter
CCNA2 Promoter
% of Input
siCTL
siSET7/9
C
SDF4 Promoter
PAGR1 Promoter
CDK5RAP2Promoter
SLX4IP Promoter
N.S
% of Input
siCTL
siSET7/9
D
CCND1 Promoter
CCNT2 Promoter
CCNA2 Promoter
% of Input
wt
K173R
K411R
E
YY1(wt) + -
YY1(K411R) - +
H1
H2A
H2B
H4

YY1 negatively-regulated genes

Term	P-Value
GO:0007156~homophilic cell adhesion	2.02E-09
GO:0016337~cell-cell adhesion	9.43E-09
GO:0022610~biological adhesion	1.23E-04
GO:0007155~cell adhesion	1.79E-04
GO:0048666~neuron development	4.76E-04
GO:0007169~transmembrane receptor protein tyrosine kinase signaling pathway	0.001026
GO:0048667~cell morphogenesis involved in neuron differentiation	0.001533
GO:0031175~neuron projection development	0.001751
GO:0007242~intracellular signaling cascade	0.002287
GO:0030030~cell projection organization	0.002445

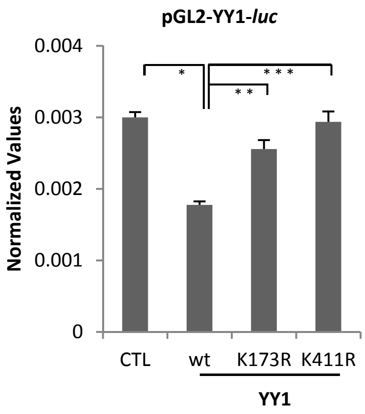
pGL2-YY1-*luc*
Normalized Values
0.004
0.003
0.002
0.001
0
*
**
***
CTL
wt
K173R
K411R
YY1
